# Determination of the geographical coordinates of the aboveground nuclear tests epicenters

**DOI:** 10.1371/journal.pone.0308920

**Published:** 2024-08-15

**Authors:** Valeriy Monayenko, Pavel Krivitskiy, Mariya Abisheva, Sergey Lukashenko, Natalya Larionova

**Affiliations:** Institute of Radiation Safety and Ecology, National Nuclear Center of the Republic of Kazakhstan, Kurchatov, Kazakhstan; University of South Carolina, UNITED STATES OF AMERICA

## Abstract

This paper presents the determination method of the exact geographical coordinates of aboveground nuclear tests (NT) epicenters based on the radioecological study results the example of the Semipalatinsk Nuclear Test Site. By testing the NT Epicenter software for determining the exact geographic coordinates of the NT centers, it was established that it is indeed possible to determine the exact coordinates of most of the aboveground NTs. Their locations are currently determined by the presence are currently determined by the presence of technogenic disturbance of the soil surface in the area of the alleged epicenter (the presence of a crater), as well as by comparing maps of radioactive contamination and a space image. The accuracy of the precise coordinates of the NT is highly dependent on the density of the auxiliary grid: the smaller the pitch of the auxiliary grid, the higher the accuracy of the NT epicenter.

## 1. Introduction

Since the advent of nuclear weapons, nuclear testing has taken place around the globe and over many decades, primarily during the second half of the 20th century. From Marshall Islands, Kiribati, and French Polynesia in the Pacific, to the desserts of Algeria, Australia, China, and southwestern United States, the steppes of Kazakhstan and elsewhere in the former Soviet Union, nuclear weapons testing has impacted people and their lands. At the same time, due to the fact that most nuclear tests were classified, the exact geographical coordinates of the epicenters of their conduct are often unknown.

The Semipalatinsk Nuclear Test Site (SNTS) is one of the world’s largest nuclear test sites. Radioactive contamination in this area has been studied by scientists of the National Nuclear Center of the [1–8]. The first testing site of the SNTS was the “Experimental Field” (EF) which was designed for atmospheric (surface and air) nuclear testing (NT). According to literature sources, it is known that 30 surface tests, including five cases where a nuclear device did not work, and 86 air nuclear tests (NT) were carried out at the EF [9]. To date, 24 nuclear test sites have been identified based on the results of the site area radiation survey conducted in 2012–2014 [10]. Identification of the NT sites and determination of the geographical coordinates was mainly based on the presence of technogenic disturbance of soil surface (the presence of craters). In the absence of any deformation of the surface layer of soil, an approximate determination of the epicenters was made by comparing maps of contamination. But in the case of overlapping radioactive contamination from several NTs it is impossible to determine exact geographical coordinates of these tests. Due to this fact, there is a need to develop a method for determining the geographical coordinates of the NTs, which allows for determination of the exact coordinates of the NT epicenters with minimum error.

Determination of the exact coordinates of the NT epicenters is very important. At present, work is being done to bring the administrative boundaries of the SNTS and its sites in line with their current radiological condition. Secondly, the presented mathematical method of determining the geographical coordinates is applicable not only to the search of the NT epicenters in the SNTS territories, but to the determination of chemical or radiation contamination epicenters in any other territories after corresponding studies.

## 2. Selection of identification parameters

### 2.1 Determination of the NT epicenter by physical (anthropogenic) disturbance

**Fig 1:**
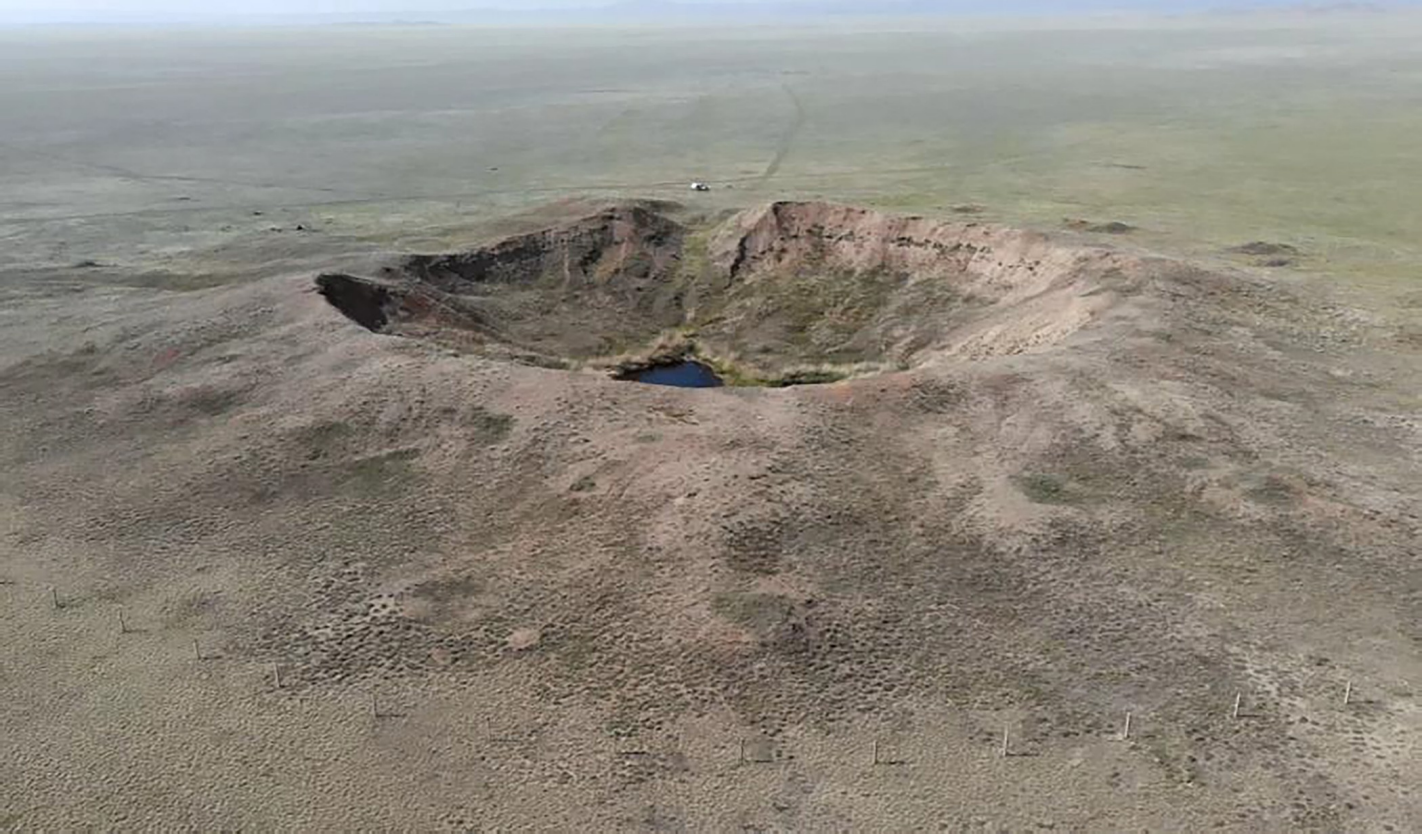
Сrater with a pronounced heap of soil [5].

As mentioned above, the easiest and most logical method of determining the epicenter of NT is the presence of technogenic disturbance of the soil surface (crater) left after the explosion. In the area of the epicenters of aboveground NTs one can often observe craters with a diameter varying from several to tens of meters with a pronounced heap of soil and slag (Fig 1).

This method is easy to use and does not require much expense, but cannot be used due to one rather large nuance. The problem is that in addition to nuclear explosions, chemical explosions have been carried out in the territory of the SNTS, which do not pose a radiation hazard, but, like nuclear explosions, leave behind an anthropogenic disturbance. In addition, based on the results of a large scale pedestrian gamma-spectrometric survey of the EF [9], it has been established that in some places of aboveground nuclear tests there are no technogenic disturbances, which is associated with conducting tests at height, testing of low power charges on the towers, as well as in the case of remediation, when the discarded soil was moved back to the crater and the epicenter area was covered with clean soil.

### 2.2 Determination of the NT epicenter by technogenic radionuclides

Considering NT as the cause of formation of technogenic radionuclides, it is necessary to note the basic physical processes behind formation of technogenic radionuclides: nuclear fission and neutron activation.

When nuclei are fissioned, fragments of nuclei with mass numbers in the intervals 85–105 and 130–150 a.m.u (atomic mass unit) are formed. Most of the technogenic radionuclides formed during the fission process have a rather insignificant half-life and only a few of them can be determined at the moment. The most indicative is ^137^Cs with a half-life of about 30 years. It should be noted that in addition to the fission fragments after a nuclear explosion, there is still a part of the unreacted nuclear fuel, which is based on the isotopes U and Pu. There is a need to note separately ^241^Am, which is a ^241^Pu β-decay product, with a half-life of more than 400 years.

In the case of neutron activation, neutron irradiation of stable isotopes of various elements contained in soil and structures occurs. The number of technogenic radionuclides formed by neutron activation is rather high, but as with the fission products, only some of the longest-lived ones have not decayed up to now: ^152^Eu– 13,5 years, ^3^H – 12,3 years, ^154^Eu– 8,6 years, ^60^Co– 5,3 years, ^155^Eu– 4,8 years.

Activation products, unlike ^137^Cs and ^241^Am, form a clear contamination plume, so the neutron field area has a clear spherical circuit. Furthermore, only small particles (<0.1 mm) are subject to transfer, which will occur only with the release of soil. Correspondingly, the products of neutron activation are suitable for determining the exact geographical coordinates of the epicenters of aboveground and near-surface nuclear tests. In order to select the most representative of the radionuclides formed by neutron ionization, results of the survey of the SNTS territory were reviewed. It was determined that ^152^Eu would be the most suitable, as it mainly prevails in the epicentral zone and thus forms a clear contour of the epicentral zone. In addition, ^152^Eu does not show migratory abilities. Thus, ^152^Eu is appropriate as the most representative to calculate the exact geographical coordinates of the epicenters of aboveground and near-surface nuclear tests.

## 3. Description and development of the method of mathematical calculation of the geographical coordinates of the NT epicenters

To calculate the exact coordinates of the NT epicenters, a method of mathematical calculation of the epicenter is proposed. This method allows one to calculate the exact geographical coordinates of the NT based on the ^152^Eu activity obtained from the results of the pedestrian gamma-ray spectrometric survey.

The general essence of mathematical calculation of the geographical coordinates of the NT epicenters is as follows:

1. selection of points of pedestrian gamma-spectrometric survey [11] is preliminarily performed in the assumed epicenter;
2. formation of limiting ranges of isolines;
3. calculation of the centers of isolines;
4. determination of the results of the calculated centers of all the specified isolines, the arithmetic mean of geographic coordinates is determined, where one coordinate, which is the epicenter of the NT, will be obtained;
5. determination of errors of the calculated epicenter of the NT are determined.

**Fig 2:**
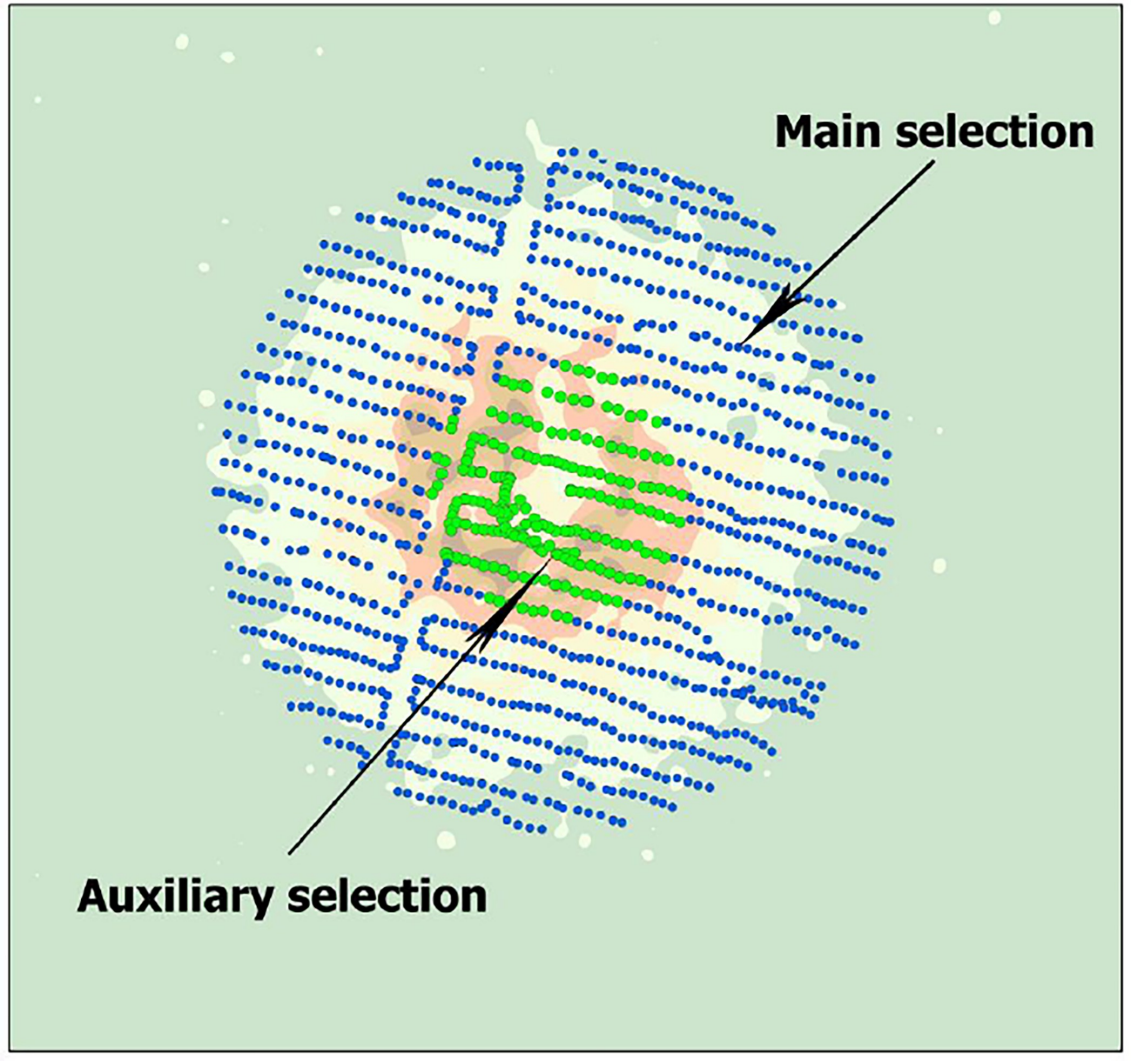
General scheme of points selection of pedestrian gamma-survey.

In order to actually develop the method of mathematical search for the epicenter of NT in the surveyed area, a selection of points of pedestrian gamma-spectrometric survey is preliminarily made on the basis of which the epicenter of NT (the main selection) will be calculated. When forming the selection, it is necessary to take into account the fact that in the area of the assumed epicenter, there is usually a pronounced technogenic disturbance of the soil cover (presence of a crater). Consequently, the points of pedestrian gamma-survey that fall into this area of the territory should be excluded from the selection, because the final result of the calculation of geographical coordinates of the epicenter of the NT will be distorted. As a selection of the epicentral zone (auxiliary selection), where the epicenter of the NT will be determined, as well as for the convenience of data processing, a selection of points of pedestrian gamma-spectrometric survey in a third of the radius of the entire "focus" of ^152^Eu activity is preliminarily made (Fig 2).

For the mathematical calculation of the epicenter of the NT, a limiting range of the radionuclide (isoline) is preliminarily specified. Isoline is a symbol on a map, which is a line at each point of which the measurable quantity has the same particular value [12]. For convenience, it is possible to divide value from a minimum to a maximum into 10 equal parts where we will receive 10 limiting ranges of isolines.

Mathematical calculation of the epicenter is performed for each previously specified isoline, and accordingly, for each of them, its own point of the proposed epicenter will be obtained. Mathematical calculation includes several operations [13,14]:

1) determination of the average distance between all points from the main selection relative to the selected point in the auxiliary selection (the determination is made for all points in the auxiliary selection).

Distance between two points A1(*x*_1_*;y*_1_) and A2(*x*_2_*;y*_2_) in the rectangular coordinate system is expressed as a formula:

$$d=\sqrt{{\left(x_{2}-x_{1}\right)}^{2}+{\left(y_{2}-y_{1}\right)}^{2}};$$ (1)

where: *d*–distance; *x*, *y*–coordinates of points.

The average distance can be calculated by formula of arithmetic mean:

$$\bar{x}=\frac{1}{n}\sum_{i=1}^{n}x_{i}=\frac{1}{n}(x_{1}+\cdots {+x}_{n})=\frac{x_{1}+\cdots +x_{n}}{n};$$ (2)

where: *x*_*n*_–variable (calculated distance of d); *n*–number of variables.

2) Determination of the difference with the average distance of all points relative to each point from the auxiliary selection.

*r* difference of *a* and *b* numbers is a result of subtracting *a* from *b*, is indicated as *a(r)* and is expressed as a formula:

r=a−b; (3)

where: *r*–difference; *a–*point distance; *b–*average distance of all points.

3) Average difference is calculated by Formula 2. The point with a minimum difference will be the assumed epicenter of the chosen isoline.
4) To determine the error of the point of the assumed epicenter, it is necessary to calculate the value of *х* (i.e. the distance from the overall result to each center of isoline), get its values: *x1*, *x2*, *х3*, *…*, *хn*. The best approximation to the true value of the calculated measurable quantity of *х* is the arithmetic mean of the calculated values:

$$x^{-}=(x1+x2+\cdots +xn)/n;$$ (4)

The degree of variation in the results of calculation and the random error can be estimated by the range of the average deviation of the results from the average value:

$$\Delta x^{-}=|x^{-}-x1|+|x^{-}-x2|+\cdots +|x^{-}-xn|)/n;$$ (5)

where *хi*—i-th (any, some) value of the obtained measurable quantity; *x*^−^–average arithmetic value calculated by Formula (4); *n*–number of measurements, i.e. the number of isoline centers found (e.g., 4–8, 8–12, 12–16…).

Based on the results of the calculated centers of all the specified isolines, the arithmetic mean of geographic coordinates is determined, where one coordinate, which is the epicenter of the NT, is obtained.

To develop the method of mathematical search for the epicenter of the NT, NT epicenter software (SW) was developed. The Object Pascal (Delphi) object-oriented programming language of the Embarcadero RAD Studio XE8 Professional application was used for coding. The main use of this language is the development of applied software.

## 4. Application example of the developed method

The developed method and software in general were tested in the area with a known epicenter of the NT, namely in the “P-2” site which has signs of aboveground NT (crater with a heap of soil).

**Fig 3:**
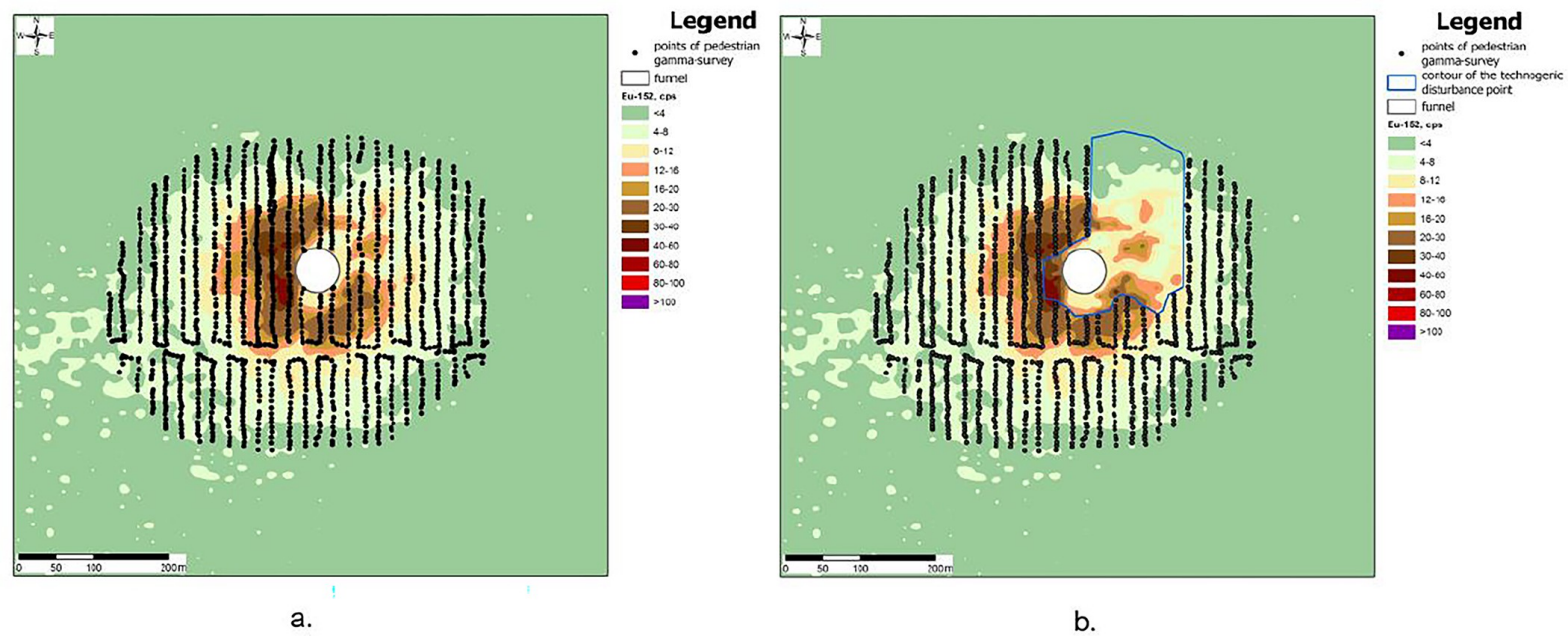
Selecting points of pedestrian gamma-survey a) general selection; b) exclusion of points in the technogenic disturbance zone.

Before starting the search for the epicenter of the NT, a selection of pedestrian gamma-spectrometric survey points was determined in the study area. Since there is a crater in the study area and there are the places with pronounced technogenic disturbance of the soil surface, the points of pedestrian gamma-survey in this area were excluded from the selection (Fig 3).

**Fig 4:**
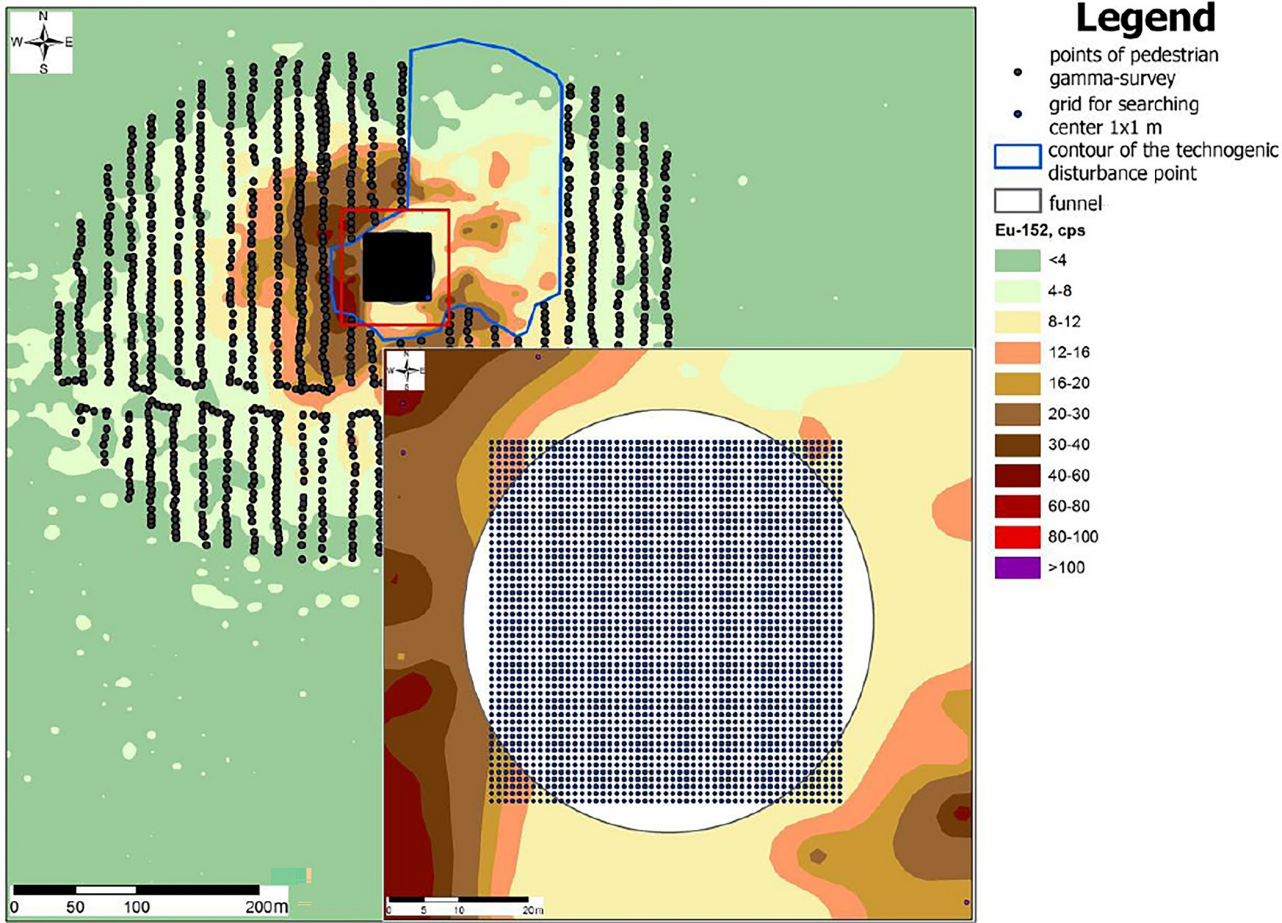
1х1 m auxiliary net.

To increase the accuracy of the search for the epicenter of the NT, an additional grid with 1x1 m pitch was superimposed in place of the assumed epicenter, the coordinates of which were used as an auxiliary selection (Fig 4). The new grid is used only as an auxiliary grid for the software calculation of the NT epicenter.

After the selection was formed with the help of the developed software the calculation of geographical coordinates of the centers of the specified isolines was carried out, namely the isoline centers of radionuclide activity were calculated: 8–12, 12–16, 16–20, 20–30 cps. Activity values for 152Eu of less than 8 cps were excluded from the calculation, as the results of the pedestrian gamma-spectrometric survey show that there is a trace extending in the north-west direction, presumably formed due to the fallout of a small fraction formed as a result of the NT and meteorologically transferred to a small distance from the NT epicenter.

Accordingly, the exact coordinates of the NT epicenter were determined on the basis of the calculated geographical coordinates of the isoline centers.

**Fig 5:**
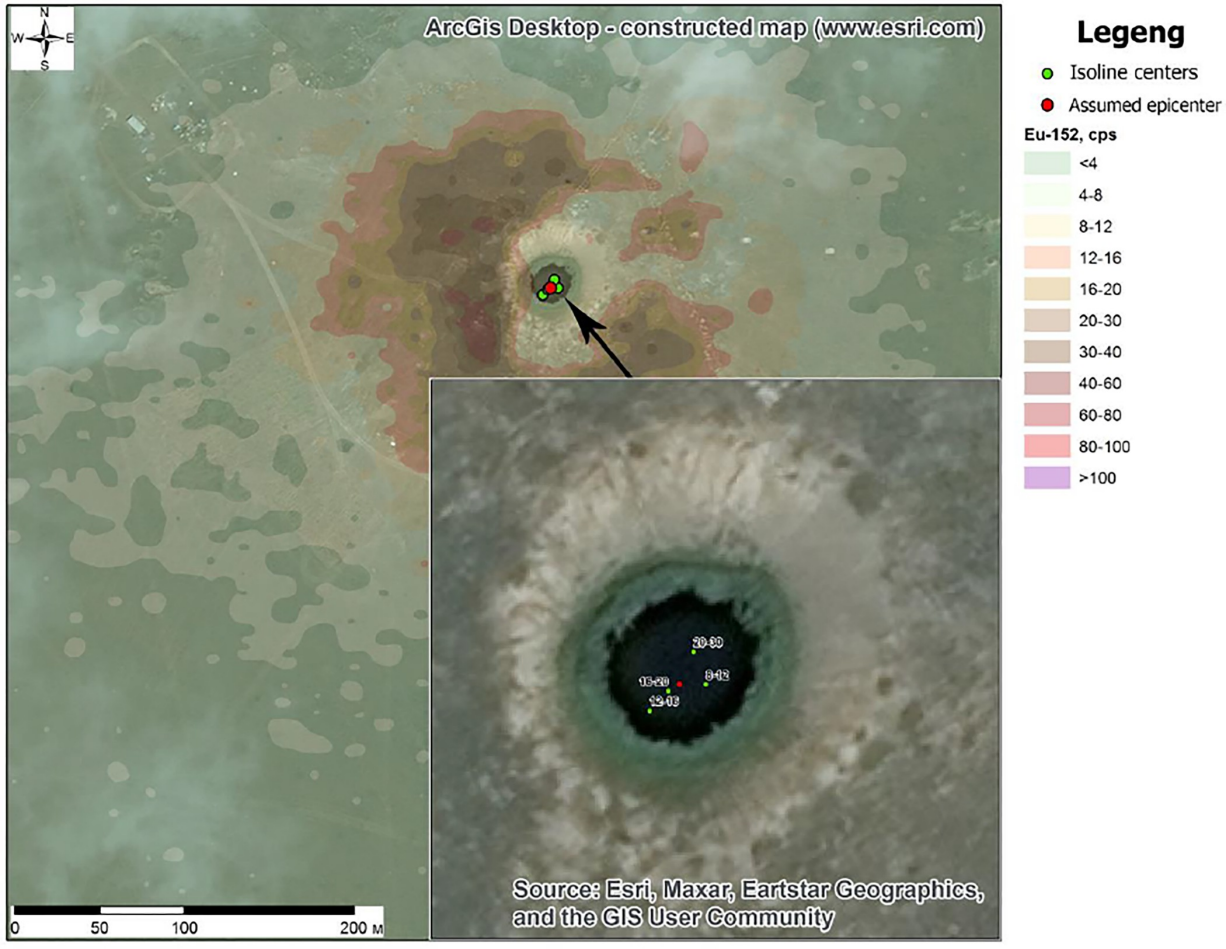
The result of the NT epicenter calculation.

To visualize the results of the epicenter calculation, the ArcGis for Desktop software (GIS platform) was used, in which the result was combined with the space image and radionuclide areal distribution map with 70% transparency, where it is clearly seen that the result of the software coincided with the spatial position of the crater. As a result, we conclude that the algorithm of the epicenter calculation works correctly (Fig 5).

## Conclusion

As a result of testing the NT epicenter software for determining the exact geographic coordinates of the NT centers it was established that it is indeed possible to determine the exact coordinates of aboveground nuclear tests using this method. This is currently determined by the presence of technogenic disturbance of the soil surface in the area of the alleged epicenter (the presence of a crater), as well as by comparing maps of radioactive contamination and a space image.

When forming the selection necessary for the calculation of the exact epicenter of the NT, special attention should be paid to the presence of technogenic disturbance of the soil surface and, accordingly, the points entering this area should be excluded from the selection, otherwise it may lead to a significant distortion of the results. Testing of the software for calculation of the exact geographical coordinates of the epicenter of NTs was performed in the area with a known epicenter of NTs, and the result of the calculation coincided with the actual location of the test (with the location of the crater) with a minimum error of about one meter.

The accuracy of the precise coordinates of the NT is highly dependent on the density of the auxiliary grid: the smaller the pitch of the auxiliary grid, the higher the accuracy of the NT epicenter. Accordingly, the developed software allows to determine the exact geographical coordinates of the NT without using special expensive GIS software, and has high efficiency and accuracy in calculating the geographical coordinates of the epicenters of ground-based NT by the indicator (activity of the ^152^Eu activation product), obtained during the large-scale pedestrian gamma-ray spectrometric survey at the “Experimental Field” testing site.
